# Direct current (DC) initiated flocculation of *Scenedesmus dimorphus*

**DOI:** 10.1007/s11356-025-36298-3

**Published:** 2025-04-02

**Authors:** Noor Haleem, Jiahui Yuan, Seyit Uguz, Serdar Ucok, ZhengRong Gu, Xufei Yang

**Affiliations:** 1 Department of Agricultural and Biosystems Engineering, South Dakota State University, Brookings, SD 57007 USA; 2 Institute of Environmental Sciences and Engineering, National University of Sciences and Technology, Islamabad, 44000 Pakistan; 3 Department of Biosystems Engineering, Faculty of Agriculture, Bursa Uludag University, Bursa, 16059 Turkey; 4 Faculty of Agriculture, Kahramanmaras Sutcu Imam University, Kahramanmaras, Turkey

**Keywords:** Microalgae, Flocculation, Harvesting, Electric double layer, Polarization

## Abstract

Despite their superior efficiency, chemical flocculants introduce foreign chemicals, such as metals, into harvested microalgae, posing downstream application challenges. To overcome this, a simple direct current (DC) initiated flocculation technology is proposed for microalgal harvesting. This method applies a DC electric field across titanium plate electrodes to promote microalgal cell aggregation by polarizing their electrical double layer. *Scenedesmus dimorphus* cultivated in Bold’s Basal Medium was tested under various voltage gradients (58, 116, 174, and 233 V/m) and energizing times (20, 40, and 60 min with DC applied), resulting in up to 94% flocculation efficiency based on dry algal biomass, significantly higher than controls. Microbubbles formed due to water electrolysis, but their impact on algal cell separation was minimal. Unlike electrocoagulation, DC-initiated flocculation uses inert electrodes and, thus, introduces fewer Fe or Al ions. This study is anticipated to facilitate research on electrochemically assisted algae harvesting and processing technologies.

**Supplementary Information:**

The online version contains supplementary material available at 10.1007/s11356-025-36298-3.

## Introduction

Due to their high yield and efficient photosynthesis, microalgae are widely regarded as a promising feedstock material to produce biofuels and value-added products such as pharmaceuticals and cosmetics. However, challenges persist in harvesting microalgal biomass from suspended culture systems. These difficulties arise from factors, such as the small size of algal cells, their strong affinity for water, low density, and negative cell surface charge (Xu et al. 2023). The expensive process of harvesting microalgal biomass poses a major obstacle, accounting for a significant portion of overall production costs (Muhammad et al. 2021). Another challenge lies in foreign materials introduced during microalgal harvesting, e.g., chemical flocculants for enhanced gravitational or mechanical separation. These challenges impede the feasibility of large-scale production of microalgae as a renewable feedstock material (Fayad et al. 2017). To mitigate these challenges, there is a need for a cost-effective and contamination-free harvesting method.

Various methods have been employed for harvesting microalgae, including physical techniques such as filtration, centrifugation, and floatation (Tan et al. 2020). Chemical methods involve the use of coagulants, flocculants, and adsorbents like magnetic nanoparticles, as well as pH adjustment as the pH of the growth medium significantly impacts the performance of these chemicals (Ummalyma et al. 2023). However, the use of chemicals raises concerns about impurities such as metals in obtained algal biomass. The presence of polymeric flocculants (e.g., polyacrylamide, PAM) also has the potential to alter the carbon profile of microalgae, limiting their range of applications (Fasaei et al. 2018). Electrocoagulation (also referred to as electro-flocculation) is another chemical harvesting method of interest (Li et al. 2020). Specifically, electrocoagulation employs anodes like aluminum (Al), iron (Fe), magnesium (Mg), copper (Cu), and zinc (Zn) that are gradually consumed during the harvesting process. For example, Shuman et al. (2016) investigated the effectiveness of microalgae harvesting using electrocoagulation-flocculation in a continuous flow reactor, applying DC across nickel and aluminum electrodes. The use of aluminum electrodes generated Al^3+^ and OH^−^ ions, leading to the formation of 'sweep flocs’ with a significant surface area. However, electrocoagulation releases metal ions like Al^3+^ that precipitate together with microalgal cells, posing challenges for downstream processing, especially in pharmaceutical applications (Krishnamoorthy et al. 2021).

**Fig. 1:**
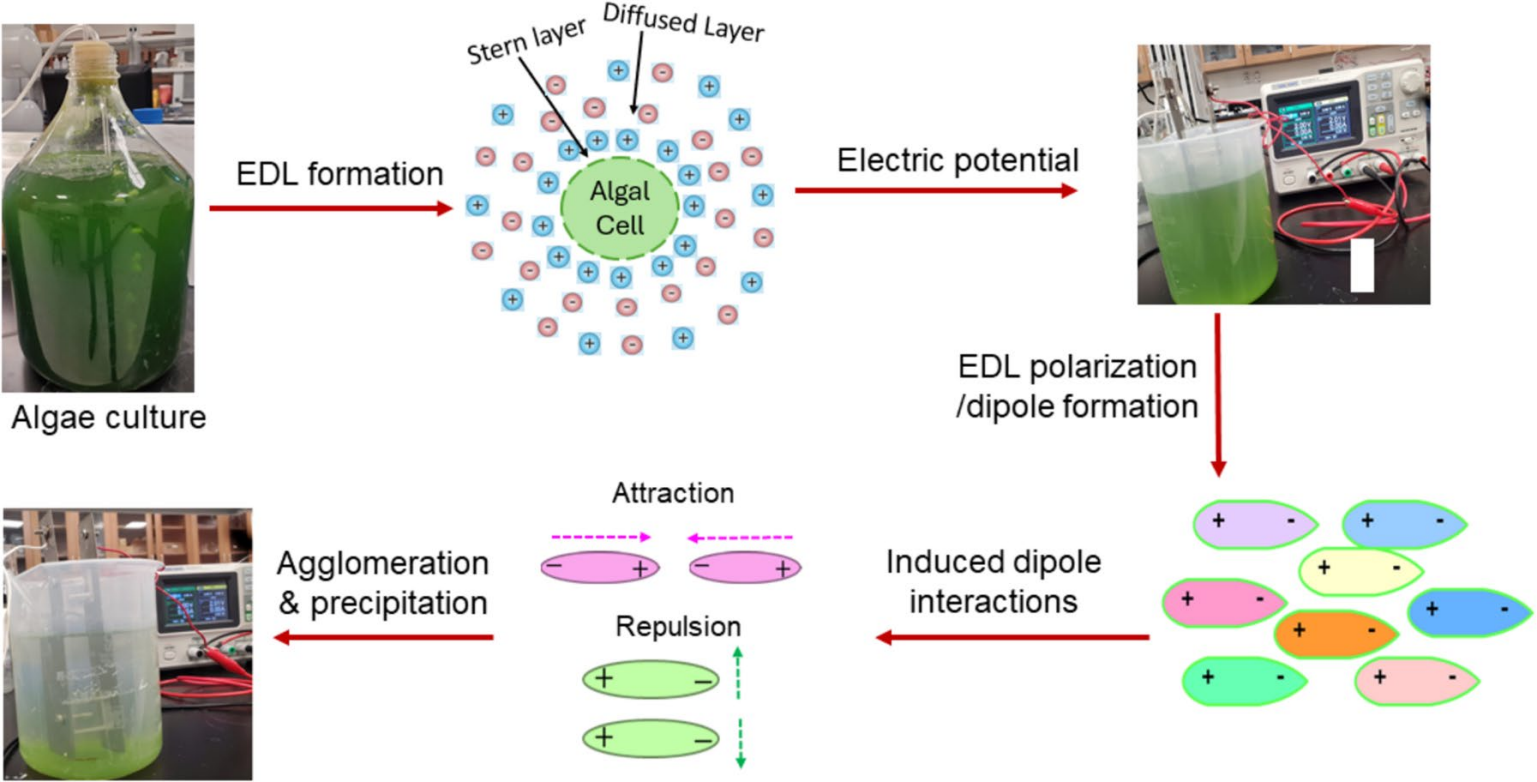
A possible mechanism of DC-initiated microalgae flocculation

This study presents a simple DC-initiated flocculation technology for microalgal harvesting. It involves the application of a DC voltage across two parallel plate inert electrodes to polarize (deform) the electric double layer (EDL) surrounding suspended microalgal cells. The polarization leads to the formation of electric dipoles and therefore induced dipole interactions, including both attraction and repulsion (Fig. 1) (Shih et al. 2015). The electrostatic attractive forces facilitate the collision and subsequent coalescence of microalgal cells, thereby initiating the process of flocculation. A similar approach has been well-established for destabilizing colloidal suspensions (Dobnikar et al. 2013). In addition to induced dipole attractions, the electrohydrodynamic convection of suspended particles in a DC field may also play a role in particle assembly (Dommersnes et al. 2013), potentially enhancing flocculation efficiency. For water treatment, the concept of DC-initiated flocculation was first introduced by a group of researchers in Japan, employing carbon electrodes (Mori et al. 2019). However, their research focused on clay-laden synthetic wastewater or mineral dressing wastewater. To our knowledge, this technology has not been explored for microalgae suspensions or organic wastewater treatment. Our investigation demonstrated the viability of employing DC-initiated flocculation for the harvesting of *Scenedesmus dimorphus*, a lipid-rich freshwater microalga with little metal contamination. This simple method eliminates the use of chemical additives and reactive electrodes, thus offering potentially reduced harvesting costs.

## Materials and methods

### Cultivation of microalgae

The cultivation followed the same protocol as reported in Osabutey et al. (2023). *Scenedesmus dimorphus* strain UTEX 1237 was cultivated using Bold’s Basal Medium (BBM) at a pH range of 7.0 ± 0.5. Initially, autoclaved BBM was blended with *Scenedesmus dimorphus* seeds. The culture was subsequently transferred to 20 L Pyrex glass bottles, with periodic addition of autoclaved BBM and deionized water. Continuous aeration was maintained at a flow rate of 0.5 L/min per liter of the algal culture, accompanied by a light intensity of 60–70 µmol m^−2^ s^−2^ using white, fluorescent lamps.

### Flocculation experiments

The microalgal suspensions obtained from the aforementioned step were set still for at least 24 h, allowing large microalgal cells or cell agglomerates to precipitate. The liquid portion (with a volume of 250 mL) was diluted with deionized water to approximately 100 mg/L dry algal biomass (DAB) and then transferred to a 500-mL beaker equipped with two Grade 2 pure titanium plates serving as electrodes (MSE Supplies LLC, Tucson, AZ). Subsequently, a DC voltage of 5, 10, 15, or 20 V (equivalent to a voltage gradient of 58.1, 116.3, 174.4, or 232.6 V/m) was applied across the electrodes for varying durations of 20, 40, 60, and 120 min (energizing time). During the duration of DC treatment, the microalgal suspension was stirred using a 40-mm magnetic stir bar at 250 RPM. After the DC treatment, the suspension remained undisturbed for 60 min, allowing flocs to settle down, before being submitted for microalgal concentration determination.

**Fig. 2:**
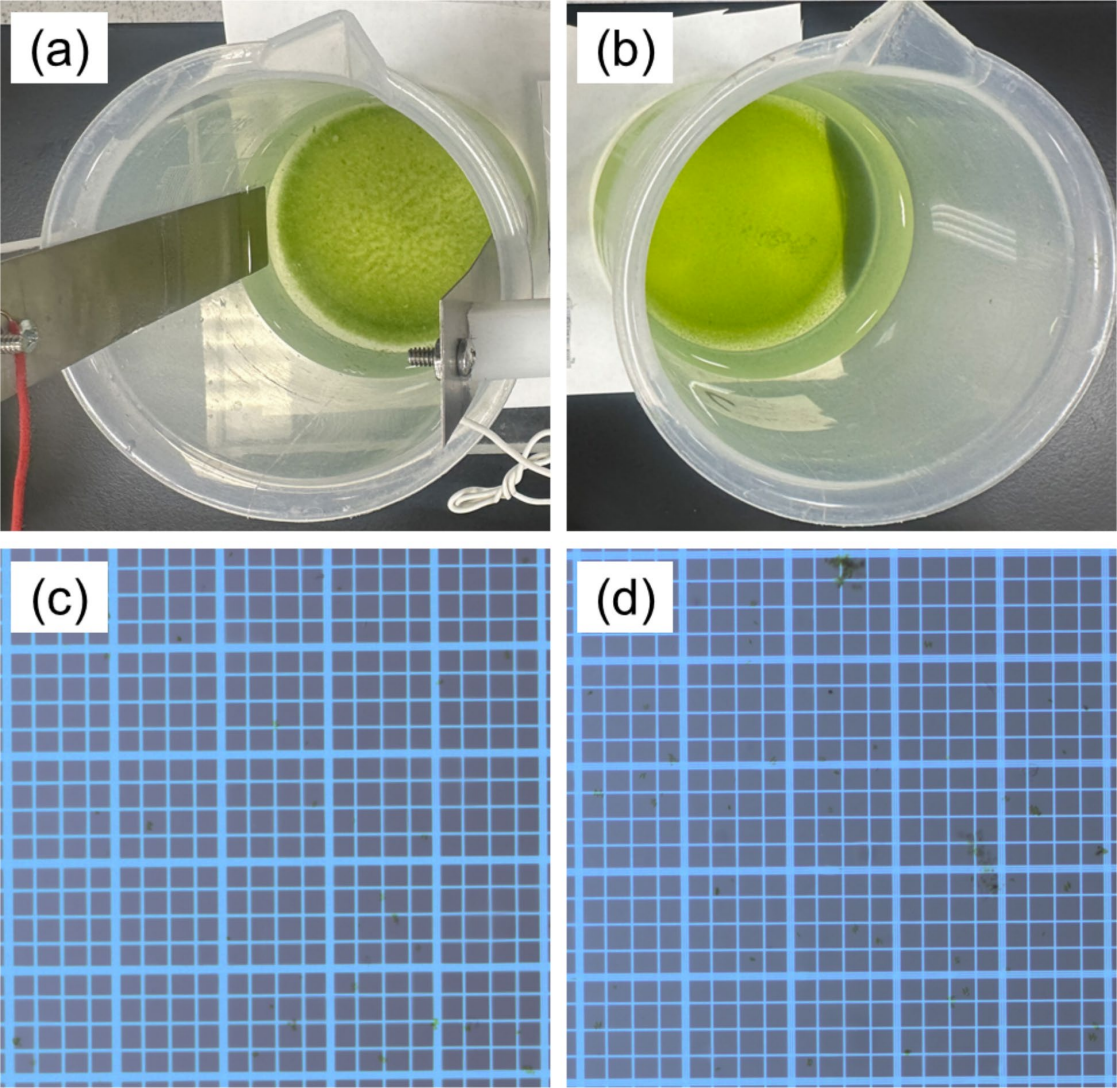
**a** Photos of a treated sample (with a voltage of 15 V) versus **b** a control sample (without voltage applied) after jar testing. **c** Microscopic images of supernatant samples of the treated versus (**d**) the control sample. Algal cell counting results are available in the Supplementary material

Before each experiment, the electrode surface was rinsed with hydrochloric acid and deionized water to remove surface deposits. The spacing between the two parallel plate electrodes was 8.6 cm and each electrode had a submerged area of approximately 15 cm^2^ (6 cm × 2.5 cm). Four beakers were employed in each run of experiments, including a control sample (0 V; stirred but without DC applied) and three replicate samples (with DC applied). The flocculation efficiency was averaged from triplicate DAB measurements of each replicate sample. The formation of flocs was observed in DC-treated samples, as illustrated in Fig. 2 and Fig. S1. After 60-min gravitational settling, supernatants were sampled approximately 15–20 mm below the liquid surface.

### Algal concentration determination

The determination of DAB concentrations (mg/L) in the supernatants followed the protocol outlined in Osabutey et al. (2023). The experiment was conducted on a jar tester (VELP JLT6, VELP Scientific, Inc.; Deer Park, New York). Flocculation efficiency was calculated as the ratio of the DAB concentrations after versus before flocculation. The optical density of the supernatant was measured using a spectrophotometer at 670 nm (OD_670_). A calibration curve was established by correlating OD_670_ readings with DAB concentrations. The cell counts of *Scenedesmus dimorphus* were determined using a Neubauer hemocytometer under an Olympus CX41 LEEDS optical microscope (Olympus Corp., Tokyo, Japan). ImageJ software was employed to facilitate the counting of microalgal cells within hemocytometer squares, with algal cell counts (cells/mL) averaged from nine squares for each sample.

### Comparison with chemical flocculation and electrocoagulation

To facilitate comparison, we conducted chemical flocculation and electrocoagulation experiments alongside DC-initiated flocculation. We choose a single Alum dosage and the same initial concentration of algal biomass to assess the effectiveness of chemical flocculation in DAB reduction. Alum (Al_2_(SO_4_)_3_), a widely used chemical flocculant in water treatment, was added to the microalgal suspension at a dosage of 5 mL of 1% solution (196 ppm). The same dosage was adopted in a previous study on the flocculation of *Chlorella ellipsoidea* (Grima et al. 2013). The experiments were conducted using a jar tester, following the methodology outlined by Haleem et al. (2023). For electrocoagulation, the titanium anode was replaced with an aluminum plate of identical size, and a DC voltage of 15 V (174.4/m) was applied for 60 min. Microalgal sediments collected from the aforementioned flocculation experiments and a control experiment were centrifuged to remove excess water before being digested for aluminum analysis, aimed at confirming the potential purity benefits of our proposed method. The digestion process followed a similar protocol described by Memić et al. (2014). In brief, approximately 3.5 g of wet algal biomass was weighed and transferred into a 50-mL centrifuge tube. A 6-mL mixture of 70% HNO_3_ and 37% HCl (in a 2:1 ratio) was added to each tube, followed by the addition of 1 mL of 30% H_2_O_2_. Each sample was digested for 24 h and then heated at 95 °C for 1 h. After cooling to room temperature, deionized water was added to the digested sample to bring the volume up to 25 mL. The samples were sent to a commercial analytical lab for Al analysis using a Thermo Scientific iCAP Pro XPS ICP-OES (Thermo Fisher Scientific Inc., Waltham, MA). The content of Al in microalgae was expressed as mg Al per g of wet algal biomass (mg/g). After drying, the samples were also analyzed for Fe using a Varian ES-715 ICP-OES (Varian Inc., Palo Alto, CA).

### Zeta potential analysis

The zeta potential (ζ; mV) of the microalgal cells was determined using a Litesizer 500 Zetasizer (Anton Paar, Ashland, VA) at a pH of 6.7, which is representative of the BBM, and no pH adjustment was applied. Before the analysis, the suspension was diluted with deionized water to raise transmittance to > 85%. The measurement results were averaged from triplicate analyses.

## Results and discussion

### Effects of voltage and energizing time

The flocculation efficiency increased with the voltage applied across the titanium electrodes. After 60-min of DC treatment (energization), the efficiency rose from 69.4 ± 0.2% at 5 V to 94.3 ± 0.3% at 20 V (Table 1). All values were greater than the DAB removal in control samples (no DC voltage applied), indicating elevated microalgal flocculation with the application of DC. For colloidal suspensions, a higher DC electric field leads to enhanced polarization of the EDL (i.e., reorganization of surface charges) of colloidal particles, resulting in greater induced dipole interactions (Shih et al. 2018). The polarization, influenced by charged particle orientation and electric field strength, primarily occurs within an EDL’s diffuse layer (Shih et al. 2015), which includes the slip plane where a zeta potential is defined. The applied DC electric field must be strong enough to overcome initial electric potentials in the diffuse layer (which are tied to the zeta potential) to induce dipole formation. We speculate that a similar explanation applies to microalgal suspensions despite their greater size than colloids.

**Table 1:** Flocculation efficiency at varying voltages and energizing times

DC voltage (V)	Energizing time (min)	DAB reduction (%)	
		Treatment	Control^A,B^
5	20	31.0 ± 1.1	26.6 ± 0.7
	40	65.1 ± 0.8	38.6 ± 0.6
	60	69.4 ± 0.7	53.4 ± 0.2
	120	66.5 ± 1.4	N/A
10	20	46.9 ± 0.8	37.4 ± 0.4
	40	55.4 ± 2.1	43.3 ± 0.5
	60	70.3 ± 0.7	45.5 ± 0.4
	120	77.6 ± 0.9	N/A
15	20	68.4 ± 0.6	41.3 ± 0.6
	40	76.0 ± 0.8	54.2 ± 0.2
	60	86.6 ± 1.1	64.8 ± 0.8
	120	84.9 ± 1.7	N/A
20	20	83.4 ± 1.9	34.8 ± 0.4
	40	90.5 ± 1.1	55.1 ± 0.6
	60	94.3 ± 0.8	63.6 ± 0.4
	120	92.1 ± 0.4	N/A

^A^The standard deviation values for the control samples were calculated from triplicate DAB analyses of each sample. The differences between the various DC voltages could be attributed to the different conditions of the algal suspensions employed for flocculation experiments

^B^No original data records were found for 120-min control samples

Notably, the control samples exhibited considerable DAB reduction due to microalgae precipitation, even after pre-sedimentation for 24 h. This can be attributed to several factors. First, the relatively large cell size of *Scenedesmus dimorphus* (approximately 4 µm × 10 µm, as determined by microscopic analysis) and its small zeta potential (−6 ± 1 mV at pH = 6.7) contribute to the instability of its suspensions. In contrast, the zeta potential of *Chlorella vulgaris* is around −18 mV at pH = 7.0, as reported in Procházková et al. (2012). Secondly, stirring of the control samples can cause algal cells to collide and aggregate, leading to the precipitation of some of the cells. Thirdly, diluting algal suspensions with deionized water to ~ 100 mg/L DAB can alter water chemistry (e.g., nutrients and ionic strength), further destabilizing the suspensions. These factors underscore the importance of including control samples in this study.

The flocculation efficiency also increased as the energizing time rose from 20 to 60 min (Table 1). The same trend was affirmed through microscopic counting of microalgal cells (Table S1 and Fig. S2). Further elongating the energizing time to 120 min, however, resulted in a slight decrease in the flocculation efficiency for the DC = 5 V, 15 V, and 20 V scenarios. This is possibly attributed to cell electroporation during elongated electric field exposure, as noted by Joannes et al. (2015). To mitigate the risk of cell lysis, we decided not to exceed DC = 20 V and t = 60 min for further experiments, following the guidance offered by a previous study on electroporation or electro-permeabilization (Goettel et al. 2013). Both the treatment and control samples were continuously stirred during the energizing time. Stirring is aimed to hydrodynamically facilitate collisions among microalgal cells, and it is also an essential step for flocculation testing using a jar tester. Without stirring, the DAB removal in the control samples was lower by approximately 5 to 10% (Fig. S3). Stirring could play a crucial role in DC-initiated flocculation by enabling “irreversible” collisions between two dipoles and the reorganization of surface charges on the formed aggregates, as demonstrated in Mori et al. (2019).

### Comparison with chemical flocculation and electrocoagulation

Three scenarios were compared: (1) chemical flocculation with 196 ppm Alum, (2) electrocoagulation with an aluminum electrode and DC = 15 V, and (3) DC-initiated flocculation with DC = 15 V (173 V/m), focusing on energy consumption and impurity levels. The choice of Alum as a chemical flocculant was due to its extensive application in water treatment and economic viability. An average flocculation efficiency of 77.4%, 83.7%, and 71.7% was observed in the three scenarios, respectively. Despite the superior flocculation performance of electrocoagulation compared to the other two methods, it consumed significantly more electricity (~ 40 mA or 2.7 mA/cm^2^; equivalent to 5.38 g of dry algal biomass harvested per kWh of electricity consumed [g/kWh]) than DC-initiated flocculation (~ 10 mA or 0.67 mA/cm^2^; equivalent to 18.4 g/kWh). As a side result, the electrocoagulation experiments generated considerably more gas (hydrogen) bubbles compared to DC-initiated flocculation (due to the high overpotential of oxygen and hydrogen evolution reactions on titanium electrodes). Few algal cells were separated through floatation in the DC-initiated flocculation experiments, as illustrated in Fig. 2. Furthermore, electrocoagulation resulted in the highest aluminum content (4.16 mg/g on a wet basis) in obtained algal biomass, followed by chemical flocculation (0.926 mg/g). A trace amount of aluminum (0.177 mg/g) was found in the algal sample obtained from the DC-initiated flocculation experiments, similar to the level (0.162 mg/g) in a control sample. This presence is likely due to aluminum impurities in the BBM or, to a lesser degree, titanium electrodes. Nevertheless, DC-initiation flocculation demonstrated great potential in minimizing impurities in harvested algal biomass.

The reduced electricity consumption in DC-initiated flocculation compared to electrocoagulation should not be overgeneralized. The testing condition (15 V for 60 min) was chosen based on the performance data of DC-initiated flocculation (Table 1) and may be far from optimal for electrocoagulation from both the performance and energy efficiency perspectives.

It is important to note that trace amounts of iron (≤ 0.3%wt Fe) could be present in Grade 2 titanium electrodes, and ferrous ions (Fe^2+^) released during DC treatment could potentially act as flocculants. To assess this effect, we analyzed the Fe content in a DC-initiated flocculation sample (15 V for 60 min) and a control algae sample. The Fe contents of the two samples were similar (0.371 mg/g and 0.363 mg/g, respectively, on a dry basis). The majority of the iron in the harvested algae likely came from the BBM, which contained 0.60 mg/L Fe^2+^. Therefore, the contribution of Fe2⁺ from the titanium electrodes appears to be minor. To eliminate the influence of metal ion flocculants, we recommend using acid-washed carbon elements in future studies.

## Discussion

Microalgae typically bear negative surface charges due to the deprotonation of certain functional groups like carboxylic (-COOH) on their cytomembranes, which are vital for cell viability and adherence (Li et al. 2022). The interaction of these charges with counter-ions and co-ions in the surroundings leads to the formation of an EDL, which consists of a densely packed, rigid Stern layer and a loosely packed diffuse layer. Zeta potential characterizes the surface potential of a charged particle at the slip plane situating within the diffuse layer, and it is a key factor governing the stability of particle suspensions (Zhang et al. 2018).

As aforementioned, DC-initiated flocculation operates through the polarization of the EDL (i.e., surface charge reorganization), leading to the formation of induced dipoles when a DC electric field is strong enough (Shilov et al. 2000). These dipoles align themselves in response to the reorganized surface charges. In a DC electric field, particles with dipole moments tend to align with field lines due to electrical torque (Baker-Jarvis and Kim 2012). When these particles align head-to-tail, they experience attractive forces, as the partially positive charge on one particle is drawn towards the partially negative charge on another particle; whereas when the particles stay head-to-head parallel, repulsive forces occur (Fig. 1) (Shih et al. 2018). Additionally, the DC electric field can alter the behavior of charged particles, causing changes in electrophoretic mobility and zeta potential (Velev et al. 2009).

Therefore, the governing mechanism of DC-initiated flocculation is distinct from the four well-known coagulation-flocculation processes, namely charge neutralization, sweep coagulation, bridging, and patch flocculation. Adding Alum or using electrocoagulation with aluminum electrodes both create large aluminum hydroxide (Al(OH)_3_) precipitates in nearly neutral pH conditions. Microalgal cells become entrapped in these precipitates and settle alongside them through sweep coagulation. Consequently, the resulting microalgal sediments from these two experiments exhibited a different feature (large, loose flocs) than DC-initiated flocculation (small, dense flocs) (Lucakova et al. 2021).

Moving forward, microalgal species (e.g., *Chlorella vulgaris*) with smaller cell size and a greater zeta potential than *Scenedesmus dimorphus* will be tested for DC-initiated flocculation. The investigation will be focused on any potential relationship between zeta potential and the minimum and optimal voltage gradient required for effective flocculation. Additional characterization of microalgal suspensions and electrodes, along with theoretical analyses, will be conducted to elucidate the underlying mechanism. Notably, without the addition of any chemicals, DC-initiated flocculation may be seamlessly interfaced with filtration (e.g., using a stainless-steel sieve as the cathode), reducing operating costs and fouling issues.

## Conclusions

This study demonstrated the viability of DC-initiated flocculation as a simple yet promising method for microalgae harvesting. It systematically investigated the effects of DC voltage and energizing time on the flocculation of *Scenedesmus dimorphus* suspensions. Under the optimal condition (DC = 20 V and *t* = 60 min), a flocculation efficiency of 94% was obtained. While slightly less efficient compared to chemical flocculation using Alum or electrocoagulation using Al electrodes, DC-initiated flocculation resulted in microalgal biomass with significantly lower Al contents. The flocculation process within a DC electric field was attributed to the polarization of the EDL of microalgal cells and subsequently induced dipole interactions.

## Supplementary Information

Below is the link to the electronic supplementary material.

Supplementary file1 (DOCX 2152 KB)

## Data Availability

Research data will be accessible upon request through South Dakota State University’s BOX service.
