# Supplementary material for: Direct current (DC) initiated flocculation of Scenedesmus dimorphus
Source: Environ Sci Pollut Res Int. 2025 Apr 2;32(17):11292–8. doi: 10.1007/s11356-025-36298-3 (PMC12014776; doi:10.1007/s11356-025-36298-3)
Supplement: Supplementary file 1 — Supplementary file1 (DOCX 2152 KB) [file 11356_2025_36298_MOESM1_ESM.docx]

Supplementary material to:

**Direct current (DC) initiated flocculation of Scenedesmus dimorphus**

Noor Haleem ^a,b^,  Jiahui Yuan^a^, Seyit Uguz ^a,c^, Serdar Ucok ^a,d^, ZhengRong Gu^a^, Xufei Yang ^a,*^,

^a^ Department of Agricultural and Biosystems Engineering, South Dakota State University, Brookings, SD 57007, USA

^b^ Institute of Environmental Sciences and Engineering National University of Sciences and Technology, Islamabad 44000, Pakistan

^c^ Department of Biosystems Engineering, Faculty of Agriculture, Bursa Uludag University, Bursa 16059, Turkey

^d^ Faculty of Agriculture, Kahramanmaras Sutcu Imam University, Kahramanmaras, Turkey

* Corresponding author; Tel: +1 605 688 5662; Email: [xufei.yang@sdstate.edu](mailto:xufei.yang@sdstate.edu)

**
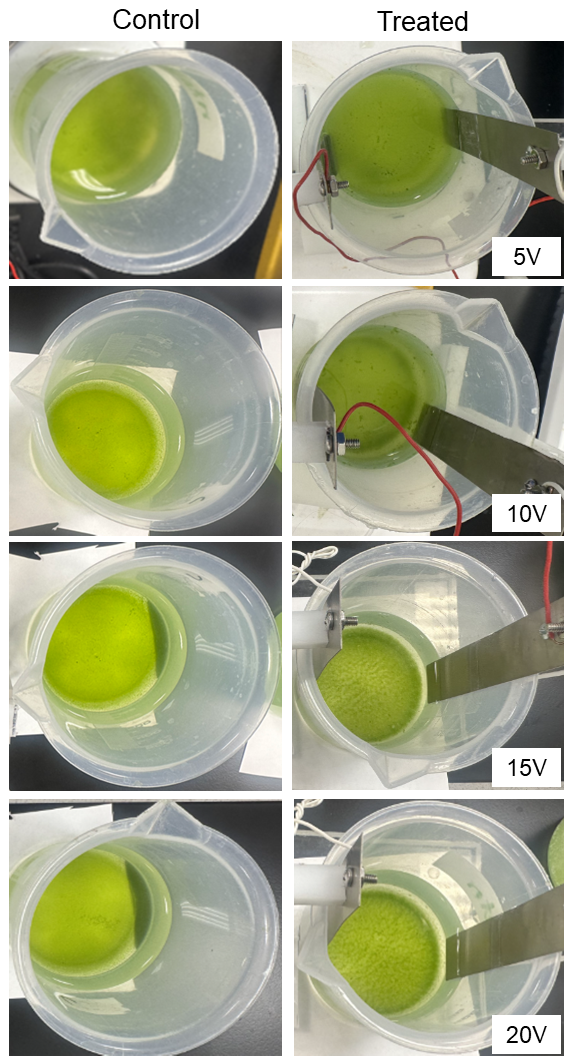
**

**Fig. S1.** Photos of DC-treated versus control samples. The formation of flocs was clearly seen in the treated samples. No significant bubble formation or floatation was observed in these samples.


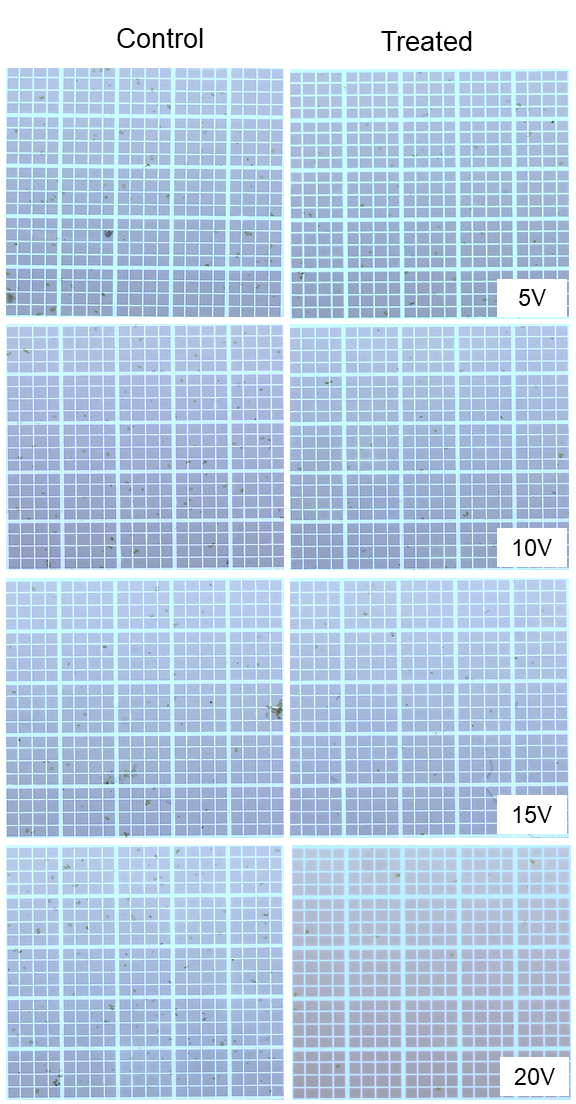


**Fig. S2.** Microscopic images of DC-treated versus control samples. Supernatants from these samples were subjected to microscopic algal cell counting. Significantly more algal cells were observed in the control samples.


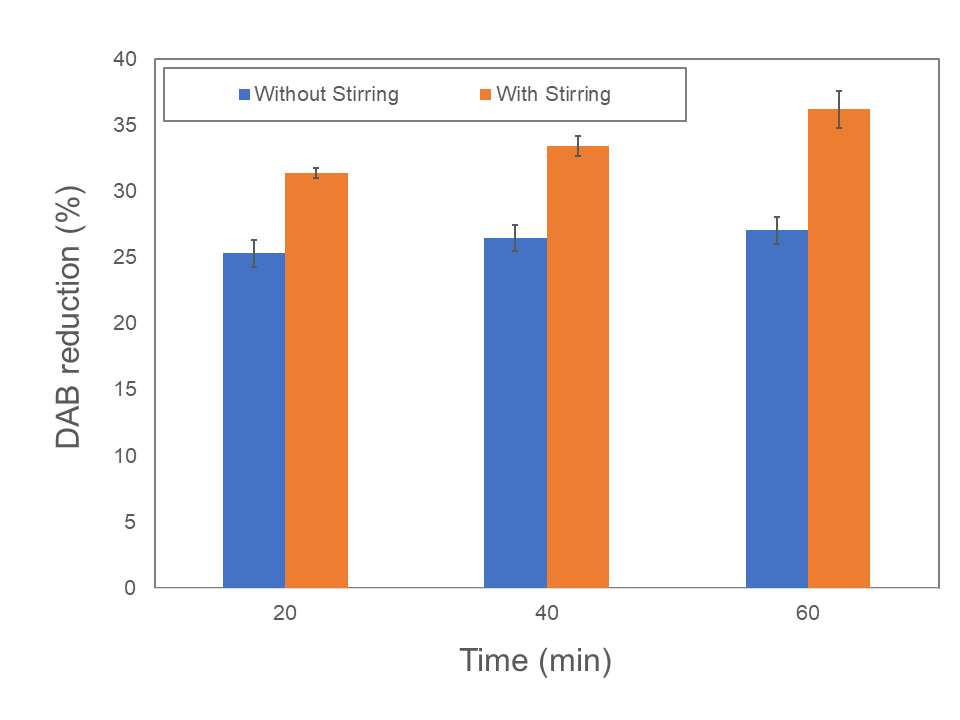


**Fig. S3.** DAB reduction due to gravitational settling when no voltage was applied: with versus without stirring.

**Table S1.** Algal cell counts in the supernatants of control versus treatment samples.*

| DC voltage (V) | Treatment time (min) | Supernatant algal cell counts (cells/cm^3^) |
| --- | --- | --- |
| 5 | 20 | 5.0 × 10^5^ |
|  | 40 | 4.8 × 10^5^ |
|  | 60 | 3.3 × 10^5^ |
|  | Control* | 7.4 × 10^5^ |
| 10 | 20 | 5.9 × 10^5^ |
|  | 40 | 4.6 × 10^5^ |
|  | 60 | 3.6 × 10^5^ |
|  | Control* | 7.9 × 10^5^ |
| 15 | 20 | 5.5 × 10^5^ |
|  | 40 | 2.1 × 10^5^ |
|  | 60 | 1.2 × 10^5^ |
|  | Control* | 8.4 × 10^5^ |
| 20 | 20 | 3.0 × 10^5^ |
|  | 40 | 6.0 × 10^4^ |
|  | 60 | 4.0 × 10^4^ |
|  | Control* | 8.2 × 10^5^ |

* The data for control samples were obtained from 60-min experiments.
